# Relation between Gastric Cancer and Protein Oxidation, DNA Damage, and Lipid Peroxidation

**DOI:** 10.1155/2013/543760

**Published:** 2013-12-24

**Authors:** Yongsheng Ma, Lin Zhang, Shengzhong Rong, Hongyan Qu, Yannan Zhang, Dong Chang, Hongzhi Pan, Wenbo Wang

**Affiliations:** ^1^The First Affiliated Hospital of Harbin Medical University, No. 199 Dongdazhi Street, Nangang District, Harbin, Heilongjiang 150001, China; ^2^Public Health School, Mudanjiang Medical College, No. 3 Tongxiang Street, Aimin District, Mudanjiang, Heilongjiang 157011, China; ^3^The Third Affiliated Hospital of Harbin Medical University, No. 150 Haping Road, Nangang District, Harbin, Heilongjiang 150081, China; ^4^Public Health School, Harbin Medical University, No. 157 Baojian Road, Nangang District, Harbin, Heilongjiang 150081, China

## Abstract

*Objects.* The aim of this study is to evaluate protein oxidation, DNA damage, and lipid peroxidation in patients with gastric cancer and to investigate the relationship between oxidative stress and gastric cancer. *Methods*. We investigated changes in serum protein carbonyl (PC), advanced oxidation protein products (AOPP), and 3-nitrotyrosine (3-NT) levels, as indicators of protein oxidation, serum 8-hydroxydeoxyguanosine (8-OHdG), as a biomarker of DNA damage, and malondialdehyde (MDA), conjugated diene (CD), 4-hydroxynonenal (4-HNE), and 8-ISO-prostaglandin F_2*α*_ (8-PGF) in serum, as lipid peroxidation markers in gastric cancer (GC) patients and healthy control. *Results*. Compared with control, a statistically significant higher values of 8-OHdG, PC, AOPP, and 3-NT were observed in the GC patients (*P* < 0.05). The products of lipid peroxidation, MDA, CD, 4-HNE, and 8-PGF, were significantly lower in the GC patients compared to those of control (*P* < 0.05). In addition, the products of oxidative stress were similar between the Helicobacter pylori positive and the negative subgroups of GC patients. *Conclusions*. GC patients were characterized by increased protein oxidation and DNA damage, and decreased lipid peroxidation. Assessment of oxidative stress and augmentation of the antioxidant defense system may be important for the treatment and prevention of gastric carcinogenesis.

## 1. Introduction

Gastric cancer (GC) is one of the most frequent diseases in human population. It is the fourth frequent cancer and the second most common cause of deaths from cancer in the world, accounting for a large proportion of cancer cases in China in recent years [1]. The pathogenesis of GC is not understood completely. Nutritional, microbial, and genetic factors acting in a multistep and multifactorial process have been proposed [2], where oxidative stress was involved in the possible mechanisms of GC development.

Reactive oxygen species (ROS) are the normal product of a variety of essential biochemical reactions and the generation of ROS is an unavoidable consequence of aerobic life. Normal level of ROS has physiological functions, whereas it is toxic to the cells at high level. Overproduction of ROS through either endogenous or exogenous insults is harmful to living organisms and is termed oxidative stress [3].

Oxidative stress can damage cellular macromolecules, leading to DNA and protein modification and lipid peroxidation [4]. Oxidative stress produced by free radicals has been associated with the development of several diseases such as cardiovascular, cancer, and chronic inflammation [3–7]. It is also known that ROS could be excessively formed in chronic diseases of the gastrointestinal tract and toxic to normal cells, which might contribute to the increased risk of cancer [6, 7], but the precise mechanisms are still remaining to be elucidated.

The aim of this study is to investigate the relationship between oxidative stress and GC. We used spectrophotometric assay and enzyme-linked immunosorbent assay (ELISA) to detect the levels of lipid peroxidation products such as malondialdehyde (MDA), conjugated dienes (CD), 4-hydroxynonenal (4-HNE), and 8-Iso-prostaglandin F_2*α*_ (8-PGF) and measured the concentration of protein damage products including advanced oxidation protein products (AOPP), protein carbonyl (PC), and 3-nitrotyrosine (3-NT) in patients with gastric cancer and healthy control subjects. In addition, we determined 8-hydroxydeoxyguanosine (8-OHdG) content, the DNA damage produced in GC patients, and healthy control, too. In the present study, we provide evidence for the first time indicating the relationship between protein carbonyl and the risk of gastric cancer.

## 2. Material and Methods

### 2.1. Subjects

This is an age-and sex-matched case-control study. Thirty patients with newly diagnosed GC are recruited for the experiment group, among whom 17 patients were positive for Helicobacter pylori (Hp^+^) and 13 negative (HP^−^). Diagnosis of gastric cancer was confirmed by histopathological examination and histopathology. Tumors were classified according to their predominant histological pattern into intestinal (*n* = 13), diffuse (*n* = 6), and adenocarcinoma (*n* = 11). The pathologic stage (TNM) was determined according to the newly revised classification of the American Joint Committee on Cancer (AJCC) and the International Union Against Cancer (UICC). Seven patients had AJCC Stage II tumors, and ten patients had AJCC Stage III tumors. The remaining thirteen patients had UICC Stage III or IV gastric adenocarcinoma. Urease test and histopathologic examination were carried out to determine the infection of Helicobacter pylori (HP). If the urease test and histology were positive, the diagnosis of HP infection was confirmed. If both tests were negative, the patient was considered Hp^−^. Exclusion criteria included subjects who had been taking antioxidant supplements and regularly used acetylsalicylic acid and other nonsteroidal anti-inflammatory drugs. Other exclusion criteria included diabetes mellitus, hypertension, arteriosclerosis, severe liver disease, cataract, renal failure, and dialysis. All patients were recruited from the Third Affiliated Hospital of the Harbin Medical University. Both groups belonged to the same ethnic group.

Thirty healthy, age-matched subjects, who came to the same hospital for an annual checkup, were also included as controls. The control subjects were in good health, as determined by a medical history questionnaire, physical examination, and normal results of clinical laboratory tests. None of the control subjects were taking any medication. All subjects gave written informed consent to participate in this study.

### 2.2. Blood Sampling

Venous peripheral blood samples (10 mL) were collected from each subject after 12 h of overnight fasting. The samples were placed on ice and centrifuged at 3500 rpm and 4°C for 15 min, and the supernatants were stored at −80°C and determination was accomplished within 3 months. All samples from each patient were run in the same assay.

### 2.3. Laboratory Analysis

AOPP were quantified as described by Witko-Sarsat et al. [8]. PC concentrations in plasma were measured with spectrophotometric assay described by Reznick and Packer [9]. CD and MDA were measured by spectrophotometric assay [4]. Serum 8-OHdG was measured with ELISA method following the maker's instructions (Highly Sensitive 8-OHdG Check, Japan Institute for the Control of Aging, Fukuroi, Shizuoka). 3-NT (Hycult Biotechnology B.V.), 4-HNE, and 8-PGF (Cell Biolabs, Inc.) were measured with ELISA following the maker's instructions. Briefly, 50–100 *μ*L of serum was added into each well of the microtiter plate and incubated for 2 h at 37°C. Secondary antibody was added to the plate and incubated at 37°C for 1 h, the unbound enzyme-labeled secondary antibody was removed, and then the antibodies binding with the plate were identified using a substrate that contained 3,3′,5,5′-tetramethylbenzidine. Absorbance was measured using a spectrophotometric plate reader (BIO-RAD) at wavelength of 450 nm. Quantification of the 8-OHdG, 3-NT, 8-PGF, and 4-HNE were done by comparing the optical densities of each sample to that of an internal standard known at various concentrations.

### 2.4. Other Biochemical Parameters

Blood glucose, triglycerides, and cholesterol were determined using routine clinical chemical assays.

### 2.5. Statistical Analysis

Data are presented as mean ± SD. All experimental data in this study were statistically analyzed with SAS 9.13. The statistical significance was evaluated using unpaired Student's *t*-test. Results were considered significant when *P* < 0.05.

## 3. Results

### 3.1. Baseline Characteristics

The clinical characteristics of the GC patients and control subjects are shown in Table 1. Age and gender of the patients were not significantly different from those of the controls. Initial clinical laboratory test values, such as glucose, cholesterol, triglycerides, and blood pressure, were also not significantly different between the two groups. What is more, all the test values were in normal range.

### 3.2. Results of Protein Oxidative Damage and DNA Damage Products in Serum

As shown in Table 2, the concentration of AOPP in GC patients was increased significantly than that of control (25.75 ± 5.75 versus 22.29 ± 5.27; *P* < 0.05). Serum 3-NT also significantly increased in GC patients compared with normal subjects (117.75 ± 37.12 versus 92.85 ± 14.47, *P* < 0.01); so was serum PC (2.16 ± 0.68 versus 1.73 ± 0.75; *P* < 0.05) and serum 8-OHdG (16.34 ± 8.30 ng/mL versus 12.29 ± 5.72 ng/mL, *P* < 0.05).

### 3.3. Results of Products of Lipid Peroxidation in Serum

As shown in Table 3, there was a significant decrease in serum MDA in cancer patients (5.07 ± 1.89 nmol/mL) compared with normal subjects (6.32 ± 1.88 nmol/mL, *P* < 0.05). Serum conjugated dienes in cancer patients have also a significant decrease than those in controls (0.534 ± 0.239 versus 0.739 ± 0.239, *P* < 0.01). So was 4-HNE (13.93 ± 7.50 versus 18.13 ± 7.95, *P* < 0.05) and 8-PGF (30.11 ± 12.65 versus 38.32 ± 10.82; *P* < 0.01).

### 3.4. Results of Oxidative Stress Products in Helicobacter Pylori Infection Positive and Negative Subgroups

We divided the 30 patients into 2 groups according to HP infection. As shown in Table 4, products of oxidative stress were similar between the HP infection positive and negative subgroups of the gastric cancer patients (*P* > 0.05).

## 4. Discussion

Gastric carcinoma is one of the most common neoplasms in the world. There are a lot of pathological factors, such as ROS, involved in the process of cancer initiation and progression, as evidenced by the fact that oxidative stress plays an important role in the molecular mechanism of gastric cancer [3, 4, 6, 7]. Exposure of proteins to reactive oxygen or nitrogen species results in modification of amino acid residues, altering the protein structure and function [10, 11]. Biomarkers of protein oxidation are often applied when a battery of markers of oxidative stress status is being studied. İn this study, we detected the contents of PC, AOPP, and 3-NT, which have been applied as biomarkers of protein oxidation. Although proteins are major targets for oxidative and nitrative damage in vivo, it is widely recognized that oxidation of proteins plays an essential role in the pathogenesis of cancers [10].

Oxidation of proteins can lead to the formation of oxidized amino acids and altered amino acid side chains containing reactive carbonyls [12]. The major product of peroxynitrite attack on proteins is an addition of a nitro group to the orthoposition of tyrosine residues to produce nitrotyrosine. 3-NT has been used as a biomarker of nitrative damage in vivo [13, 14]. The presence of 3-NT in some carcinomas was previously reported [15]. In our study, serum 3-NT level was significantly higher in the GC patients. This result is constant with other researchers' findings. Goto et al. [16] and Bancel et al. [17] found that 3-NT levels are elevated in gastric cancer patients.

Protein carbonyl is the most popular biomarker for severe oxidative protein damage [18]. It can be generated through oxidative cleavage of proteins by either the *α*-amidation pathway or by oxidation of glutamyl side chains, leading to the formation of a peptide in which the N-terminal amino acid is blocked by an *α*-ketoacyl derivative [10]. The previous studies showed that cancer patients have higher levels of PC [4, 19]. But Hoque et al. found that there was no significant change in prostate cancer patients of serum PC in a large nested case-control study [20]. In this study, we found increased PC levels in gastric cancer. Our results show that PC is a potential biomarker available for assessing oxidative stress in gastric cancer.

AOPP, products of the action of free radicals on proteins, were described by Witko-Sarsat et al. [8] for the first time. AOPP is defined as dityrosine containing cross-linked protein products and are considered to be reliable biomarkers to estimate the degree of protein oxidation [21]. They are elevated in patients with renal insufficiency and they reach the highest levels in patients after renal replacement therapy [22]. Increased levels were also found in cancer patients where they correlated with markers of oxidative stress [23, 24]. We found that the level of AOPP was significantly higher in GC patients. In accordance with our findings, Noyan et al. [25] showed that AOPP concentration was significantly higher in the serum specimens from gastric cancer than those from the normal control.

In the present study, we have determined the protein oxidation products of 3-NT, AOPP, and PC. All the protein oxidation products were significantly higher in GC patients. This means that there was severe protein oxidation damage in gastric cancer patients.

ROS can cause strand breaks and base modifications in DNA. For example, oxidation of guanine residues to 8-OHdG is an oxidized nucleoside of DNA that is the most frequently detected and studied DNA lesion. It has been regarded as a novel biomarker for oxidative DNA damage in vivo and appears to play a crucial role in mutagenesis. Elevated levels of 8-OHdG were demonstrated in some carcinomas, including gastric cancer [15, 26–28]. Farinati et al. [27] and Hahm et al. [28] showed a significant increase of 8-OHdG in HP-positive and -negative gastric cancer patients. Interestingly, the levels of 8-OHdG in gastric mucosa have been shown to decrease substantially after the eradication of HP infection. In our research we also found that there was increased serum 8-OHdG in gastric cancer. Nevertheless, the greater oxidative DNA damage in the GC, as a possible result of impaired antioxidant activity, implies an important role for oxygen free radicals in stomach carcinogenesis. Therefore, the contents of 8-OHdG in serum could act as a sensitive biomarker for GC patients, too.

Lipid peroxidation is initiated by free radical attack on membrane lipids, generating large amounts of reactive products, which have been implicated in tumor initiation and promotion. In this study, we selected a series of products of lipid peroxidation, MDA, CD, 8-PGF, and 4-HNE, which are widely used, sensitive, and applicable for large scale studies.

MDA is a product of the breakdown of mainly unsaturated fatty acids into their essential chains through oxidation, which is the most studied free radical reaction. But the data reported in the literature on MDA levels in different human cancer types remains controversial. Some researchers reported that lipid peroxidation increased and MDA level advanced in cancer patients. Arivazhagan et al. [29] reported increased erythrocyte MDA levels in patients with gastric cancer. Khanzode et al. [30] and Bakan et al. [31] reported increased plasma MDA levels in gastric cancer patients compared with those in control subjects. However, some other reports are not consistent with these findings. Punnonen et al. [32] found increased serum MDA levels but decreased tissue MDA levels in human breast cancer. Chang et al. [4] and Biasi et al. [33] reported decreased serum MDA levels in human colon cancer. Murugan et al. [34] reported significantly decreased serum MDA levels in N-methyl-N′-nitro-N-nitrosoguanidine (MNNG)-induced gastric carcinogenesis in rat model. Inconsistent with some but consistent with other findings, our results show a decrease of serum MDA levels in patients compared with those in control subjects [30, 31].

CD is the initial formation of the lipid peroxide. Murugan et al. [34] reported significantly decreased serum CD levels in MNNG-induced gastric cancer in rats. Chang et al. [4] revealed significantly decreased serum CD levels in colorectal cancer patients. 4-HNE is the major *α*, *β*-unsaturated aldehydes produced by membrane lipid peroxidation, and it can be a potential indicator of lipid peroxidation. Murugan et al. [34] also reported significantly decreased serum 4-HNE levels in MNNG-induced gastric carcinogenesis in rat model. İn the present study, we found that there were decreased CD and 4-HNE in serum in GC patients compared with control subjects.

The prostaglandin, 8-PGF, is an isoprostane that is produced by the nonenzymatic peroxidation of arachidonic acid in membrane phospholipids [35]. 8-PGF is a major isoprostane that is chemically stable and measurable in biofluids, and is a reliable biomarker for assessing the rate of lipid peroxidation of subjects Other than the widely appreciated biomarker, Camphausen et al. [36] found it unattainable to detect an increased 8-PGF in the urine of patients with prostate cancer compared with normal controls. We found that the level of 8-PGF was significantly lower compared with control. As far as we know, our study represents the first report to test the relationship between serum 8-PGF, and the risk of gastric cancer.

Considering that there are some contradictory results of lipid peroxidation reported in cancer, in this study we select a series of different products of lipid peroxidation, including MDA, CD, 4-HNE, and 8-PGF, to investigate the relationship between GC and lipid peroxidation. We found, for the first time, the lipid peroxidation products prone to the same declining trend, with all the products of lipid peroxidation being decreased significantly in serum of gastric cancer patients compared to healthy control. We propose that the decreased levels of MDA, CD, 4-HNE and 8-PGF in GC patients may relate to tumor aggressiveness which is in line with the hypothesis proposed by Slater et al. [37], who assumed that rapidly dividing cells tend to set an oxidant-antioxidant status favorable to their growth. Rapidly proliferating tumor cells show resistance to lipid peroxidation and overexpress antioxidant enzymes. Peroxidation is deleterious for cell proliferation through the production of reactive oxygen species, with the exact mechanism remaining unraveled. Further studies are needed to conclusively determinate the correlation between the lipid peroxidation and cancer.

Helicobacter pylori related gastritis is characterized by increased free radical production and peroxidative damage [38, 39]. Production of ROS by gastric cells and phagocytes induced by HP has been shown in vitro, and increased levels of ROS in the gastric mucosa have been verified in HP-infected patients [40, 41]. A positive association has been demonstrated between HP infection and gastric adenocarcinoma with increased oxidative stress [42].

We therefore investigated whether HP infection was also related to a progressive accumulation of oxidative mutagenic events that could play a major role in gastric carcinogenesis. The 8-OHdG levels were significantly higher in GC than in the control group, but significant difference was not detected between HP-positive and -negative samples in spite of slightly higher 8-OHdG levels in the HP-positive GC patients. The result is in line with the findings of Farinati et al. [43] and Noyan et al. [25], where the level of AOPP was increased significantly in the gastric cancer patients as compared with nongastric cancer patients. However, there were no significant differences between the anti-HP IgG positive and negative patients with gastric cancer. And we also found that the level of AOPP was not significantly different between the HP-positive and -negative gastric cancer patients. Goto et al. [16] demonstrated that 3-NT in the gastric mucosa was significantly higher in the HP-positive group than in the HP-negative group. But our results showed that there was no significant difference in oxidative stress parameters between the HP-positive and -negative gastric cancer patients. These contradictory results suggest that the relationship between HP infection and gastric adenocarcinoma with increased oxidative stress still remains unclear. Although approximately half of the world population are infected with HP, a cancerous outcome of infection is very rare. Hence, whether HP infection is a major causative factor for GC remains questionable according to the discrepancies among the current literature.

## 5. Conclusions

In summary, the results obtained here confirm that oxidative stress is involved in gastric cancer, though there was no significant difference in oxidative stress parameters between the HP-positive and -negative gastric cancer patients. We found that the gastric cancer patients had increased levels of protein oxidation and DNA damage versus control subjects, but decreased lipid peroxidation products. The mechanism remains to be fully revealed. It would be worthwhile to further study the correlation between these biomarkers and levels of lipid peroxidation and GC. Assessment of oxidative stress and augmentation of the antioxidant defense systems may be important for the treatment and prevention of gastric cancer.

## Figures and Tables

**Table 1:** Demographic and clinical characteristics of patients and controls.

Factors	Control	Patients
*N*	30	30
Age (years)	62.5 ± 8.4	61.4 ± 7.7
Sex (F/M)	17/13	19/11
Cholesterol (mmol/L)	4.92 ± 0.63	5.06 ± 0.68
Triglycerides (mmol/L)	1.06 ± 0.33	1.13 ± 0.41
Glucose (mmol/L)	4.62 ± 0.87	4.56 ± 0.65
Systolic blood pressure (mmHg)	124.5 ± 9.0	124.7 ± 9.3
Diastolic blood pressure (mmHg)	76.5 ± 6.6	77.8 ± 7.3
Smoking habit		
Current smokers	8	6
Nonsmokers	16	14
Ex-smokers	6	10
Alcohol intake (Y/N)	16/14	17/13
Physical exercise (Y/N)	18/12	10/20
History of family (Y/N)		6/24

Data are means ± SD.

**Table 2:** Serum levels of protein oxidation and DNA damage products.

Group	AOPP (*μ*mol/mL)	3-NT (ng/mL)	Protein carbonyl (nmol/mL)	8-OHdG (ng/mL)
Control	22.29 ± 5.27	92.85 ± 14.47	1.73 ± 0.75	12.29 ± 5.72
Patients	25.75 ± 5.75*	117.75 ± 37.12**	2.16 ± 0.68*	16.34 ± 8.30*

Data are means ± SD. *P < 0.05 compared to controls, **P < 0.01 versus healthy subject.

**Table 3:** Serum levels of lipid peroxidation products.

Group	MDA (nmol/mL)	8-PGF (pg/mL)	4-HNE (nmol/mL)	CD (OD, 233 nm)
Control	6.32 ± 1.88	38.32 ± 10.82	18.13 ± 7.95	0.739 ± 0.239
Patients	5.07 ± 1.89*	30.11 ± 12.65**	13.93 ± 7.50*	0.534 ± 0.233**

Data are means ± SD. *P < 0.05 versus healthy subject, **P < 0.01 versus healthy subject.

**Table 4:** The results of oxidative stress products in H. pylori infection patients.

	HP infection Negative (Hp^−^)	HP infection Positive (Hp^+^)
*n*	13	17
MDA (nmol/mL)	4.90 ± 1.49	5.20 ± 2.18
CD (OD, 233 nm)	0.551 ± 0.192	0.521 ± 0.265
4-HNE (nmol/mL)	14.65 ± 9.84	13.37 ± 5.35
8-PGF (pg/mL)	28.74 ± 8.11	31.15 ± 15.43
AOPP (*μ*mol/mL)	25.99 ± 6.01	25.56 ± 5.72
PC (nmol/mL)	2.13 ± 0.81	2.18 ± 0.58
3-NT (ng/mL)	114.49 ± 44.56	120.25 ± 31.51
8-OHdG (ng/mL)	15.36 ± 7.72	17.09 ± 8.88

Data are means ± SD.
